# Effect of Maternity Units' Organizational Levels on Maternal Birth Satisfaction: A Multicentric Cohort Study

**DOI:** 10.1111/birt.12909

**Published:** 2025-03-03

**Authors:** Simona Fumagalli, Antonella Nespoli, Maria Panzeri, Laura Antolini, Elisabetta Colciago, Anna Adami, Matilde Maria Canepa, Elsa Del Bo, Raffaella Ferrara, Paola Agnese Mauri, Angelo Cagnacci, Marcello Ceccaroni, Carmen Dattolo, Giovanna Esposito, Massimo Piergiuseppe Franchi, Franco Gorlero, Gianpaolo Grisolia, Francesca Grosso, Agnese Lecis, Marta Mazzeo Melchionda, Virginia Michelerio, Luana Mogavino, Chiara Ogliari, Michela Ramunno, Arsenio Spinillo, Sabrina Valletta, Patrizia Vergani, Anna Locatelli

**Affiliations:** ^1^ School of Medicine and Surgery University of Milano Bicocca Monza Italy; ^2^ Department of Obstetric Foundation IRCCS San Gerardo Dei Tintori Monza Italy; ^3^ Center for Biostatistics, Department of Clinical Medicine, Prevention and Biotechnology University of Milano Bicocca Monza Italy; ^4^ Department of Medicine and Surgery University of Verona Verona Italy; ^5^ Midwifery School, Department of Neurosciences, Rehabilitation, Ophthalmology, Genetics, Maternal and Child Health (DiNOGMI) University of Genoa Genoa Italy; ^6^ Department of Clinical‐Surgical, Diagnostic and Pediatric Sciences University of Pavia Pavia Italy; ^7^ Department of Obstetrics and Gynaecology Carlo Poma Hospital Mantua Italy; ^8^ Department of Clinical Sciences and Community Health Università Degli Studi di Milano Milan Italy; ^9^ Department of Woman, Newborn and Child Fondazione IRCCS Ca' Granda Ospedale Maggiore Policlinico Milan Italy; ^10^ Medical and Midwifery School, Department of Neurosciences, Rehabilitation, Ophthalmology, Genetics, Maternal and Child Health (DiNOGMI) University of Genoa Genoa Italy; ^11^ Department of Obstetrics and Gynecology IRCCS Sacro Cuore‐Don Calabria Hospital Negrar Italy; ^12^ Department of Obstetrics and Pediatrics Azienda Socio Sanitaria Territoriale Vimercate Vimercate Italy; ^13^ Department of Obstetrics and Gynecology University Hospital of Verona, University of Verona Verona Italy; ^14^ Obstetrics and Gynecology Unit Galliera Hospital Genoa Italy; ^15^ Department of Obstetrics and Gynecology San Martino Hospital Genoa Italy; ^16^ Department of Obstetrics and Gynecology, IRCCS Fondazione Policlinico San Matteo University of Pavia Pavia Italy; ^17^ Department of Obstetrics and Gynecology MBBM Foundation at San Gerardo Hospital Monza Italy

**Keywords:** assessment tool, BSS‐R, Italy, maternal birth satisfaction, place of birth, quality of care

## Abstract

**Introduction:**

Maternal birth satisfaction is correlated to long‐term outcomes and is influenced by the place of birth. In Italy, most births occur in hospitals. Our study aimed to assess whether the organizational level (I vs. II) of the Maternity Unit (MU) had any impact on birth satisfaction.

**Methods:**

A multicentric cohort study was conducted in 11 Italian MUs, classified as Level I (for low‐risk pregnancies or with minor complications) or Level II (for low and high‐risk women) according to organizational, structural, and technical standards. Birth satisfaction was measured using the Italian version of the BSS‐R, composed of three sub‐scales. Data analysis was performed using Stata/MP18.0.

**Results:**

Among 1642 participants, maternal satisfaction was similar in I and II level MUs (27.7 vs. 27.2; *p*‐value 0.096). Women who gave birth in an I level MU were found to have a greater Quality of care sub‐scale score compared to participants who gave birth in a II level MU (14.28 vs. 13.87; *p*‐value < 0.001). The three sub‐scales contributed differently to the total score, with a minor contribution given by the Stress Experienced (8.65/16) and the Women's Attributes sub‐scales (4.72/8).

**Conclusion:**

This study contributes to understanding how the level of the MU might impact women's birth satisfaction. Factors affecting the Women's Attributes and the Stress Experienced sub‐scales' scores should be considered to increase maternal satisfaction with birth, improving the quality of maternity services.

## 1 Introduction

Worldwide, providing high‐quality care during childbirth is one of the most important goals for health services [1]. According to the World Health Organization (WHO) all childbearing women should expect a positive birth experience as a recommended outcome, which can be achieved when the birth experience aligns with a woman's personal and sociocultural beliefs and expectations [2, 3, 4]. A positive birth experience contributes to maternal satisfaction and should be considered a key quality measure for evaluating and improving maternity healthcare services [2, 3].

The WHO defines maternal satisfaction as the extent to which service standards meet expectations [1]. It is a multidimensional and subjective concept, influenced by parents' expectations about childbirth, interpersonal aspects of care (such as the model of care, communication style, and the presence of a birth companion), personal control, and self‐efficacy [5, 6, 7, 8].

Mother's satisfaction is correlated with long‐term maternal and neonatal outcomes. A high satisfaction with the birth experience might have a positive impact on parental attitudes, including future birth choice [9]. While dissatisfaction is linked to increased rates of psychological diseases, breastfeeding difficulties, caesarean sections, and miscarriage in future pregnancies [7, 10, 11].

Birthplace is a crucial component of birth, which involves physical, emotional, cultural, and social aspects, with an impact on maternal childbirth experience [12]. Therefore, all dimensions of care, including structure, process, and outcome, have an influence on maternal satisfaction [13].

In high‐income countries the access to alternative birthplaces varies within and between countries [14]. In Italy, almost all women give birth in hospitals [15]. The Italian NHS provides universal and free of charge maternity care mostly in obstetric‐led units and in a few midwifery‐led units [16, 17]. Italian Maternity Units (MU) are classified into two levels of care (I Level and II Level) according to organizational, structural, and technical standards [18], fully described in Table 1. I Level Maternity Units (MU) provide care to pregnant women over 34 weeks who do not require maternal and neonatal high‐complex interventions, and they can choose where to give birth. II Level MUs provide care to all pregnant women regardless of their obstetric risk profile [18]. However, high‐risk women are usually referred to II level Mus.

**TABLE 1:** Organizational, Structural, and Technical standards that accurately describe I Level MU and II Level MU as defined by law [17].

	I Level MU	II Level MU
Target population	Gestational age ≥ 34 gestational week	All gestational age
	Low and Medium obstetric risk: excluding women or foetus with high‐complexity interventions	Low, Medium and High obstetric risk: including women or foetus with high‐ complexity interventions
Number of births per year	500–1000	> 1000
Organizational standards	Essential staffing 24/7: almost 2 midwives per shift until 1000 births Presence of almost a gynecologist, a pediatrician and an anesthetists	Essential staffing 24/7: Almost 3 midwives per shift if births/year < 1500; 4 if < 2000 and 5 if > 2000 Presence of almost 2 gynecologists, a pediatrician and a anesthetist
	Essential environment: almost 20 beds/1000 births 3. Labour suits 1. Operating room dedicated to obstetric emergencies 1. Delivery room dedicated to Low risk labour	Essential environment: Almost 20 beds/1000 births 3 Labour suits (4 if births/year> 2000) and 1 extra Labour suite 1 Operating room dedicated to obstetric emergencies (2 if births/year> 1500) 1 Delivery room dedicated to Low risk labour
	Essential services: 1 ultrasound scanner in the Delivery Unit Sub‐Intensive care Unit for women and newborns Laboratory tests, blood transfusion and diagnostic imaging available 24/7 Maternal Assisted Transport to Level II MU	Essential services: 1 ultrasound scanner in the Delivery Unit Intensive care Unit for women and newborns Laboratory tests, blood transfusion and diagnostic imaging available 24/7 Neonatal Assisted Transport from Level I MU Availability of additional specialist care to women (as psychological, neurological, …)
Structural standards	Adequate connection between the Maternity Ward and the Delivery Unit Almost birth‐equipment for 2 contemporary births Fetal monitor for each Labour suite Equipment for non pharmacological coping strategies Almost a room dedicated to post‐partum women observation	Adequate connection between the Maternity Ward and the Delivery Unit Almost birth‐equipment for 3 contemporary births Fetal monitor for each Labour suite Equipment for non pharmacological coping strategies Almost a room dedicated to post‐partum women observation Dedicated room to anesthetic's counseling during pregnancy
Technical standards	Periodic evaluation of function and efficiency of every technologies available	Periodic evaluation of function and efficiency of every technologies available

Both I Level and II Level MUs are distributed across Italy, but their availability can vary significantly between regions. Urban areas, particularly those with larger hospitals, are more likely to have II Level MUs to handle complex cases. This distribution ensures that all pregnant women have access to appropriate care, though there may be differences in travel distance and accessibility based on location. Efforts are ongoing to balance the distribution of these units to meet regional healthcare demands effectively.

Although recommended by the WHO [1], birth satisfaction is not included in the Italian Maternal and Neonatal Outcomes Reporting System [15].

Several studies have explored how maternal birth experience can be affected by birth environments, including spatial and social dimensions [19, 20], as well as midwifery care, one‐to‐one care, staffing level, and intrapartum interventions [7, 21, 22, 23, 24, 25].

Although the role of the quality of maternity services in ensuring a positive birth experience is strongly demonstrated [2, 3], there is a gap of knowledge about the effect that the organizational level of MU might have on birth satisfaction.

Our study aimed to investigate whether the organizational level (I vs. II Level) of the MU had any impact on birth satisfaction in Italy.

## 2 Methods

We conducted a multicentric cohort study. Women who gave birth in 11 maternity units, identified through the educational network of the north‐Italian midwifery bachelor courses, were recruited.

In 2010, the Italian Ministry of Health published the State‐Regions Agreement [18] containing 10 recommendations, including a reorganization of care for maternity hospitals and the implementation of different pathways depending on obstetric risk profile. According to organizational, structural, and technical standards defined at the national level (Table 1), five I Level MUs and six II Level MUs were enrolled. Data S1 describes the characteristics of each research site involved in the study.

Participants were recruited through a quota sampling method among women who gave birth in the MUs involved in the study from January 1, 2022, to July 1, 2022. Each I Level MU recruited 100 women, while each II Level MU enrolled 200 participants.

The inclusion criteria were: healthy women with a straightforward pregnancies without major complications (such as cardiac disease, pre‐eclampsia, haemoglobinopathies, renal disease, neurological disease, sepsis), aged between 18 and 50 years, with appropriate speaking and reading abilities, who gave birth at term (37–42 gestational weeks).

The exclusion criteria were: planned or pre‐labour caesarean section, newborn in poor condition at birth or requiring resuscitation, and foetal or neonatal death.

Women who met the inclusion criteria were invited to participate in the study at least 24 h after giving birth. Participants were informed about the study's aim and its voluntary and confidential nature. Informed consent was gained from all the participants.

Our expectation on the sample size of this observational study, considering previous data conducted in one of the participating centres [17], was about 500 women who gave birth in I Level MUs and about 1000 who gave birth in II Level MUs.

The observed sample size was a total of 1642 women. Of those, 470 gave birth in I level MUs and 1172 gave birth in II Level MUs. Considering the BSS‐R total score ranging from 0 to 40 points, our sample enabled us to detect an effect size of 0.28 standard deviations with a significance level equal to 0.05 and a power equal to 0.90.

Maternal birth satisfaction was measured by the Italian version of the Birth Satisfaction Scale Revised (I‐BSS‐R), an English self‐report scale that was translated and adapted within the Italian context [11, 26, 27]. The I‐BSS‐R is a reliable and valid tool useful for assessing maternal satisfaction with birth in the studied context [17].

The I‐BSS‐R consists of three sub‐scales: Quality of Care Provided (QC), Women's personal Attributes (WA), and Stress Experienced during labour (SE). The QC sub‐scale is made up of 4 questions that explore the support offered to women to promote their empowerment during labour, the communication's quality with health workers and the birth's environment. The WA sub‐scale is defined by 2 items regarding the perceptions of personal concerns about labour and birth and the loss of personal‐control (both these items are reverse‐coding). The SE sub‐scale is made up of 4 questions that investigate concerns for herself in both physical and psychological dimension (as possible complications), having experienced long labour and the characteristic of intrapartum experience (stress and concerns during labour). Each of the ten items has 5 points and is scored using a Likert‐type scale ranging from 0 to 4 (0 “strongly disagree”, 4 “strongly agree”), and four of them are reverse‐coded (e.g., “I found childbirth a distressing experience”) [26].

The I‐BBS‐R scores were analyzed considering both the total score and each sub‐scale [28]. The 25° centile of the I‐BBS‐R distribution (in our sample equal to 24) was used to define a new variable called “low satisfaction” to identify a group of women who, on average, were less satisfied than the study population.

Socio‐demographic characteristics, obstetric history, intrapartum variables, and maternal and postnatal outcomes were also explored.

### 2.1 Statistical Analysis

The characteristics of the entire sample and the ones within Levels of MUs were described using frequencies and percentages for categorical and discrete variables and using summary indicators (mean and standard deviation (SD)) for continuous variables. Distributions differences within levels were tested using the Chi‐square test (for categorical variables) and the *t*‐test (for continuous variables).

To assess the association between the levels of MUs and maternal satisfaction with birth, it was necessary to eliminate or reduce the confounding effects of confounding variables, which were related to the observational nature of the study. Confounding variables are those that influence the treatment (I Level MU and I Level MU) and outcome (maternal birth satisfaction). To reduce the confounding effects and investigate the association between levels of MUs and maternal satisfaction, the inverse probability of treatment weighting method (IPTW) [29] was used: it reduces or eliminates the effects of confounding to estimate treatment effects using non‐randomized data. The IPTW allowed us to create a pseudo‐population where confounding variables were balanced; the confounding variables were defined within characteristics that were temporarily antecedent to the treatment, the choice of the place of birth, and that had different distributions within MU's levels: maternal age, university degree, previous caesarean‐section and parity. The association between MU's levels and maternal satisfaction at birth (total score, each subscale, and the presence/absence of low satisfaction) was detected using balanced data from the IPTW method.

All tests performed were two‐sided, and a *p*‐value ≤ 0.05 was considered statistically significant.

Statistical analyses were performed using Stata/MP 18.0.

### 2.2 Ethical Consideration

The Local Ethics Committee approved this study before the beginning of the participants' enrollment (MSM_2019, December 23, 2019). Written Informed consent was obtained from all participants.

## 3 Results

Among the 1642 women recruited, 470 (28.6%) women gave birth in a I Level MU and 1172 (71.4%) in a II Level.

Women who gave birth in a II Level MU had higher levels of education (*p*‐value 0.01) and were mostly nulliparous (*p*‐value < 0.001), compared to women who had access to a I Level MU. The frequency of the previous caesarean section was higher across II Level MUs (*p*‐value 0.012). No differences within the antenatal care were identified between groups. Among nulliparous women who gave birth in a I Level MU, more often attended antenatal classes (*p*‐value < 0.001) (Table 2).

**TABLE 2:** Distribution of Socio‐demographic variables, obstetric history, antenatal care, maternal outcomes, and post‐natal care in the entire sample and within MU's levels.

		Overall (*n* = 1642)		I Level MU (*n* = 470)		II Level MU (*n* = 1172)		*p*
		Mean	SD	Mean	SD	Mean	SD	
Socio demographic variables								
Maternal age (years)		32.91	4.96	32.29	4.65	33.17	5.06	0.001

		*n*	%	*n*	%	*n*	%	
University degree		827	50.37	213	45.32	614	52.39	0.01
Employed		1274	77.59	353	75.11	921	78.58	0.127
Obstetric history								
Primiparous		855	52.07	199	42.34	656	55.97	< 0.001
Previous vaginal instrumental birth		39	2.38	8	1.7	31	2.65	0.257
Previous caesarean section		71	4.32	11	2.34	60	5.12	0.012
Antenatal care								
Antenatal class attending		736	44.82	227	48.3	509	43.43	0.073
Public care in pregnancy		710	43.43	187	39.79	523	44.89	0.059
Midwife presence during pregnancy care		450	27.41	128	27.23	322	27.47	0.921
Intrapartum care								
Spontaneous onset of labour		1133	69.04	345	73.4	788	67.29	0.015
One to one		1314	80.12	446	95.1	868	74.12	< 0.001
Non Pharmacological analgesia		1100	66.99	347	73.83	753	64.25	< 0.001
Intermittent auscultation of fetal hearth		409	24.94	71	15.11	338	28.89	< 0.001
Prologed active phase of first stage (≥ 12 h)		58	3.54	22	4.69	36	3.07	0.109
Augmentation with Oxytocin		559	34.09	129	27.45	430	36.75	< 0.001
Epidural analgesia		711	43.33	159	33.9	552	47.1	< 0.001
Maternal outcomes								
Mode of birth	Spontaneous	1425	86.78	409	87.02	1016	86.69	0.84
	Vacuum assisted	93	5.66	28	5.96	65	5.55	
	Caesarean section	124	7.55	33	7.02	91	7.76	
Episiotomy^a^		225	14.82	48	10.98	177	16.37	0.007
Perineal integrity^a^		353	23.25	119	27.23	234	21.65	0.02
Spontaneous delivery of placenta		1517	92.44	436	92.96	1081	92.24	0.614
Post‐natal care								
Skin to skin		1422	86.6	421	89.57	1001	85.41	0.025
First breastfeed until 1 h		1399	85.2	395	84.04	1004	85.67	0.403

^a^ Percentages calculated within women who had spontaneous or vacuum‐assisted vaginal birth (*n* = 1518).

Women who gave birth in a I level MU received a different intrapartum care compared to women who delivered in a II level MU (Table 2). One‐to‐one midwifery care, non‐pharmacological strategies to cope with pain, and skin‐toto‐skin contact were more likely to be provided in I level MUs. Moreover, lower levels of intrapartum intervention rates (epidural analgesia, oxytocin augmentation and episiotomy) were reported in I level MUs compared to the care provided in II Level MUs.

A total of 1425 women (86.79%) had a spontaneous vaginal birth, 124 (7.55%) women had a caesarean section, and 93 had an instrumental vaginal birth (*n* = 93, 5.66%). No significant differences were identified between levels of MU (Table 2).

**FIGURE 1:**
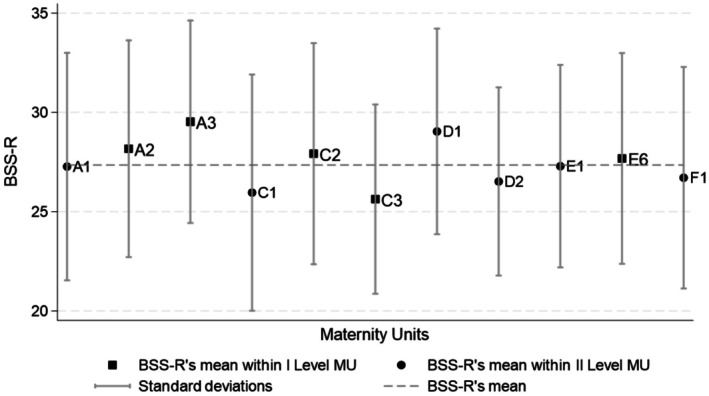
Distribution of I‐BSS‐R within Maternity Units involved in the study.

The comparison between and within levels of MUs is reported in Figure 1. The mean of the I‐BSS‐R total score was 27.35 (SD = 5.46), with higher values in women who gave birth in a I Level MU (27.92 vs. 27.12) (Table 3). Overall, the scores of the three sub‐scales contributed differently to the total score (Table 3): the mean of the QC sub‐scale was 13.98, the maximum achievable is 16; while the mean of the WA sub‐scale was 4.72, the maximum achievable is 8, and the mean of the SE sub‐scale was 8.65, the maximum achievable is 16.

**TABLE 3:** I‐BSS‐R total score and subscale in the entire sample and within levels.

	Item (*N*)	Overall (*n* = 1642)		I Level MU (*n* = 470)		II Level MU (*n* = 1172)	
		Mean	SD	Mean	SD	Mean	SD
I‐BSS‐R	10	27.35	5.46	27.92	5.39	27.12	5.48
Sub‐Scale							
Quality of care provision	4	13.98	2.02	14.26	1.86	13.87	2.07
Women's personal attributes	2	4.72	1.95	4.67	1.98	4.74	1.94
Stress experienced	4	8.65	3.24	8.99	3.29	8.51	3.21

After propensity score adjustment, maternal satisfaction was similar within women who gave birth in I Level and II Level MUs (mean 27.70 vs. mean 27.20; *p*‐value 0.096) (Table 4). Of note, the QC sub‐scale was significantly higher in women who gave birth in an I Level MU (mean 14.28; 95% CI: 14.1; 14.46) compared to women who gave birth in a II Level MU (mean 13.87; 95% CI: 13.65; 14.09) (*p*‐value < 0.001) (Table 4). No differences between levels were identified for both the WA (*p*‐value 0.159) and the SE (*p*‐value 0.164) sub‐scales (Table 4). In the overall sample, 386 women have been included in the “low satisfaction” group, with a BSS‐R total score lower than 24. The probability of being less satisfied than the average was higher in women who gave birth in II Level MUs (24.70% vs. 20.28%; *p*‐value 0.057) (Table 4).

**TABLE 4:** I‐BSS‐R (total score and sub‐scales) within I and II Level MUs and distribution of the “Low satisfaction” group (I‐BSS‐R < 25° c.tile) with balanced data (Propensity score weighting).

	I Level MU		II Level MU		*p*
	Mean	95% CI	Mean	95% CI	
I‐BSS‐R	27.7	[27.19; 28.21]	27.2	[26.61; 27.79]	0.096
Sub‐Scale					
Quality of care provision	14.28	[14.1; 14.46]	13.87	[13.65;14.09]	< 0.001
Women's personal attributes	4.6	[4.41; 4.78]	4.75	[4.54; 4.97]	0.159
Stress experienced	8.83	[8.52; 9.13]	8.58	[8.23;8.93]	0.164

## 4 Discussion

Our study is the first exploring the effect of the organizational level of the MU on maternal birth satisfaction within the Italian context, where births occur almost exclusively in hospital settings [18, 30]. The impact of birthplaces on maternal experience has been widely explored in literature [31, 32, 33]. Our study, which focused on the role of organizational, structural, and technical aspects of MUs, contributes to increasing knowledge about birth satisfaction in hospital settings.

The main strength of the present study is its multicenter nature, with eleven participating research sites, including a large sample size, which ensures the generalizability of our results for healthy pregnant women. Furthermore, the power of our study is enhanced by the use of the IPTW method [29] that minimizes or eliminates the confounders revealed by the literature [6, 17, 34], resulting in a more accurate evaluation of the impact of the organizational level on maternal birth satisfaction.

One of the main limitations of our study is the exclusion of high‐risk women and other vulnerable groups from the target population. This exclusion may affect the generalizability of our findings, especially given the significant representation of these groups in our country [35]. Therefore, future research should aim to include women with higher risk profiles to provide a more comprehensive understanding of birth satisfaction across different populations [2, 3, 36]. Including these groups in future studies may either reinforce the trends observed in low‐risk women or reveal new factors influencing maternal satisfaction, thus offering valuable insights for improving maternity care services for all women.

In addition, future research exploring Italian mothers' experiences within different birth settings, such as MLU and home, is warranted. Another interesting topic might be to investigate determinants of maternal birth satisfaction.

We found an average score of the I‐BSS‐R that falls within the range reported across similar studies [6, 24, 37, 38, 39], which is between 24.2 [24] and 31.94 [6]. When focusing on each sub‐scale, similarities and differences were found compared to the literature. The average score of our QC sub‐scale was consistent with the one reported by previous authors [6, 37, 38, 39]. When considering the average score of the SE sub‐scale, it turned out to be lower than prior evidence [6, 37, 38, 39]. In contrast, the average score of the WA sub‐scale was similar to the one reported by the same cited studies [37, 38, 39] but lower than the one evaluated in the USA study by Fleming et al. [6], who recruited women from different birth settings.

Our results showed that MU's organizational level did not affect maternal birth satisfaction, with similar I‐BSS‐R total scores within I Level and II Level MUs. This finding might be explained by a similar distribution of modes of birth observed in both I and II Level MUs. Mode of birth has been suggested as one of the factors that impact most on maternal birth satisfaction [6, 22, 34]. On the contrary, we observed that Level I and II MUs adopt different approaches to intrapartum care, with midwifery care in level I MUs being more focused on promoting normal labor and birth [16, 17, 33, 40]. Although this was not the primary aim of our study, our data indicated that women in I Level MUs were more likely to experience spontaneous labor onset, use non‐pharmacological pain relief, receive one‐to‐one midwifery care, and were less likely to undergo labor augmentation. These findings align with previous research in a similar context [35]. Moreover, existing studies suggest that healthy women giving birth in high birth volume hospitals might be more exposed to interventions such as epidural analgesia and episiotomy. This could be due to staff shortages, time pressure from simultaneous births, or the higher frequency of complicated cases, which may lead to overtreatment of low‐risk women [41, 42].

Another interesting finding is the one showing that women who gave birth in II level MUs were more likely to be included in the “low satisfaction” group, compared to participants who gave birth in I level MUs. Our hypothesis is that the abovementioned results might be linked. In fact, midwives who routinely look after both low‐and high‐risk women in II level MUs are exposed to increasing amounts of interventions that might also influence their attitude towards childbirth care. This higher perception of risk perceived by midwives could lead to a higher provision of unnecessary obstetric interventions, thus contributing to a medicalisation of birth. This might be the reason why women in II Level MUs, who were more likely to experience interventions, were also at higher risk of being less satisfied than women who give birth in a I level MU. Midwives working in I level MUs should be more familiar with a health‐promoting approach, supporting normal birth. Hence, Midwife‐Led Unit should be considered one of the evidence‐based strategies to improve several maternal and neonatal outcomes, including maternal satisfaction with birth.

The aspects mentioned above, related to midwifery practice, had an influence on the three sub‐scales of the total I‐BSS‐R score. The QL, WA, and SE sub‐scales assess different dimensions related to maternal satisfaction with birth. The comparison of each sub‐scale between I and II Level MUs enables us to deeply explore the impact of the organizational level on the birth experience.

When we considered each sub‐scale separately, the QC sub‐scale had a greater score when the birth occurred in an I level MU. Again, this finding might be explained by the hypothesis that I Level MUs are more likely to promote all aspects investigated by the QC sub‐scale, including women's empowerment, support by professional staff, and presence of a comfortable environment [26]. The literature suggests that one‐to‐one midwifery care, non‐pharmacological coping strategies and skin‐to‐skin contact, have a positive impact on the quality of care and in turn on maternal birth satisfaction [16, 33, 40]. In this regard, we found that those procedures were offered more often in I level MUs. Thus, contributing to increasing the probability of being in the “low satisfaction” group in women who gave birth in II level MUs. Unlike the QC sub‐scale, the MU's organizational level did not affect the scores of the SE and the WA sub‐scales, with mean values that were similar in both I and II Level MUs. The SE sub‐scale assesses the physical and psychological stress due to prolonged labour, vacuum‐assisted birth, and caesarean section [26]. The similar approach and adoption of these intrapartum interventions within settings could explain the absence of difference in the average scores of the SE sub‐scale between MU levels.

Moreover, the WA sub‐scale explores maternal competencies, such as women's concerns about labour and perception of control [26]. According to the literature, midwifery antenatal care and continuity models of care have a key role in counseling, they promote an ongoing individual adjustment of maternal expectation of birth that facilitates women's decision‐making, women's awareness, emotional support, and, in turn, a higher maternal satisfaction [43, 44, 45, 46]. In our study, the characteristics of the antenatal care offered to women were very similar regardless of the level of the MU where they gave birth, including the type of healthcare professionals providing the care, characteristics of the setting where the birth occurred (public or private hospitals) and rate of antenatal education attendance. In addition, the model of care provided to women during the childbearing pathway was not included as one of the criteria to define the difference between organizational levels of maternity units in the Italian State‐Regions Agreement [18]. All these considerations might explain why no significant differences were observed in the WA sub‐scale average scores between the two levels of MUs.

The average scores of the SE and the WA sub‐scales were significantly below the maximum achievable score. Based on these findings, maternity healthcare professionals should consider the items measured by both the SE and the WA sub‐scales to improve women's birth experiences. For instance, based on the knowledge that intrapartum interventions have an impact on the SE sub‐scale [26], the promotion of the normal progress of labor should be supported as a potential strategy to enhance maternal satisfaction. Again, the development of a partnership relationship between women and midwives might positively affect aspects investigated by the WA sub‐scale [43, 44, 45, 46]. The present study could be an opportunity to identify which components of care deserve attention, thus facilitating the implementation of effective strategies or interventions to promote satisfaction, regardless of the organizational standards. Most of the concepts that are embedded in the BSS‐R sub‐scales are strongly supported by both the midwifery care models of care and the philosophy promoted by Midwife Led Units (MLU) [47]. For this reason, MLU should be offered to women with straightforward pregnancies within the Italian health services. In practical terms, strategies such as continuous one‐to‐one midwifery care throughout labor, the use of non‐pharmacological pain relief methods (e.g., breathing techniques, hydrotherapy, or massage), and encouraging women to actively participate in decision‐making during labor have been shown to improve maternal satisfaction and overall birth outcomes. Evidence suggests that these approaches promote a more positive birth experience, reducing the need for unnecessary interventions. Furthermore, ensuring adequate staffing levels to allow personalized care and training healthcare professionals to adopt a woman‐centered approach are crucial for successfully implementing these strategies in everyday clinical practice.

### 4.1 Practical Implications

- Provide evidence‐based care regardless of the healthcare setting, ensuring that all women receive high‐quality, individualized care.
- Ensure adequate staffing levels, access to necessary equipment, and ongoing training for healthcare professionals to maintain high standards of care.
- Utilize validated tools for risk assessment to accurately identify low‐risk women and tailor care accordingly.
- Empower women with information about their care options and the potential benefits and risks associated with different interventions.
- Develop and distribute educational materials, offer counseling sessions, and engage women in discussions about their preferences and choices during antenatal visits.
- Advocate for policy changes to integrate MLUs within the healthcare system and ensure they are adequately funded and staffed.

By incorporating these practical strategies into clinical practice, maternal health professionals can contribute to more positive birth experiences and improve overall maternal satisfaction.

## 5 Conclusion

The present study showed that the level of the MU, defined using organizational, structural, and technical criteria, did not impact maternal satisfaction with birth. However, the classification used to define the level of an Italian MU does not consider the model of care provided to the women, which is considered to have an important influence on maternal satisfaction.

This study suggests key strategies to address high‐quality maternal and newborn healthcare, which might contribute to promoting a positive experience of birth for all women. The key role of midwifery models of care in helping women to achieve empowerment, a high perception of control, and the best health outcomes should be acknowledged. This study contributes to understanding how the level of the MU, defined by organizational, structural, and technical standards, might impact women's birth satisfaction. In addition, this research might provide the basis for future investigations regarding the role of variables affecting the I‐BSS‐R. In particular, we should focus on factors influencing the WA and the SE sub‐scales, aiming at identifying strategies or interventions to promote a positive birth experience.

This research did not receive any specific grant from funding agencies in the public, commercial, or not‐for‐profit sectors. The authors declare that there is not any conflict of interest.

## Ethics Statement

The Local Ethics Committee approved this study before the beginning of the participants enrollment (approval number: MSM_2019). Written Informed consent was obtained from all participants.

## Conflicts of Interest

The authors declare no conflicts of interest.

## Supporting information


Data S1.

## Data Availability

The data that support the findings of this study are available from the corresponding author upon reasonable request.
