# Supplementary material for: Effect of Maternity Units' Organizational Levels on Maternal Birth Satisfaction: A Multicentric Cohort Study
Source: Birth. 2025 Mar 3;52(4):641–51. doi: 10.1111/birt.12909 (PMC12612367; doi:10.1111/birt.12909)
Supplement: Supplementary file 1 — Data S1. [file BIRT-52-641-s001.docx]

**Supplementary Material**

Characteristics of Maternity Units involved in the study: number of births in 2022, number of Caesarean sections within Maternity Unit (Caesarean sections’ proportion among births in 2022), Robson classification, presence of intrapartum and childbearing period’s protocols for healthy women.

In columns “N” are reported the number of Caesarean sections in each Robson’s group and in columns “%” are reported the proportion of Caesarean sections within each Robson’s group.

I Level Maternity Units:

|  | | **A2** | | **A3** | | **C2** | | **C3** | | **E6** | |
| --- | --- | --- | --- | --- | --- | --- | --- | --- | --- | --- | --- |
| N° of births in 2022 | | 1092 | | 1087 | | 723 | | 476 | | 761 | |
| N° Caesarean sections (%) | | 157 (14.4%) | | 184 (16.9%) | | 260 (36%) | | 142 (29.8%) | | 222 (29.2%) | |
|  | | N | % | N | % | N | % | N | % | N | % |
| Robson Classification | Group 1 | 17/290 | 5.9 | 19/275 | 6.9 | 25 | N/A | 15 | N/A | 16/212 | 7.5 |
|  | Group 2 | 39/172 | 22.7 | 29/150 | 21.6 | 71 | N/A | 19 | N/A | 74/249 | 29.7 |
|  | Group 3 | 4/378 | 1.1 | 8/349 | 2.3 | 6 | N/A | 6 | N/A | 2/162 | 1.2 |
|  | Group 4 | 11/124 | 8.9 | 6/116 | 5.3 | 11 | N/A | 4 | N/A | 17/112 | 15.2 |
|  | Group 5 | 55/78 | 70.5 | 71/115 | 61.7 | 88 | N/A | 75 | N/A | 71/81 | 87.6 |
|  | Group 6 | 14/14 | 100 | 18/18 | 100 | 26 | N/A | 5 | N/A | 19/19 | 100 |
|  | Group 7 | 11/11 | 100 | 15/16 | 93.8 | 18 | N/A | 4 | N/A | 10/10 | 100 |
|  | Group 8 | 2/4 | 50 | 0/3 | 0 | 6 | N/A | 3 | N/A | 2/2 | 100 |
|  | Group 9 | 0 | 0 | 1/1 | 100 | 1 | N/A | 2 | N/A | 0 | 0 |
|  | Group 10 | 4/21 | 19.1 | 12/39 | 30.7 | 6 | N/A | 9 | N/A | 11/20 | 55 |
| Presence of intrapartum protocol for healthy women | | YES | | YES | | YES | | YES | | YES | |
| Presence of protocols for healthy childbearing women | | YES | | YES | | YES | | NO | | YES | |

II Level Maternity Units:

|  | | **A1** | | **C1** | | **D1** | | **D2** | | **E1** | | **F1** | |
| --- | --- | --- | --- | --- | --- | --- | --- | --- | --- | --- | --- | --- | --- |
| N° of births in 2022 | | 2560 | | 1620 | | 5878 | | 1568 | | 2594 | | 1986 | |
| N° Caesarean sections (%) | | 480 (18.7%) | | 581 (35.6%) | | 2460 (41.8%) | | 460 (29.3%) | | 773 (29.8%) | | 487 (24.5%) | |
|  | | N | % | N | % | N | % | N | % | N | % | N | % |
| Robson Classification | Group 1 | 33/660 | 5 | 54 | N/A | 198/1128 | 17.5 | 11/230 | 4.8 | 43/581 | 7.4 | 24/405 | 5.9 |
|  | Group 2 | 123/427 | 28.8 | 235 | N/A | 682/1446 | 47.2 | 122/256 | 47.6 | 189/501 | 37.7 | 95/365 | 26.0 |
|  | Group 3 | 8/728 | 1.1 | 6 | N/A | 65/988 | 6.6 | 17/317 | 5.4 | 9/584 | 1.5 | 4/500 | 0.8 |
|  | Group 4 | 25/234 | 10.7 | 13 | N/A | 320/990 | 32.3 | 154/318 | 48.4 | 54/2777 | 19.5 | 42/246 | 17.1 |
|  | Group 5 | 144/234 | 61.5 | 99 | N/A | 471/580 | 81.2 | 65/211 | 30.8 | 224/290 | 77.2 | 149/210 | 70.9 |
|  | Group 6 | 49/52 | 94.2 | 43 | N/A | 162/162 | 100 | 18/18 | 100 | 110/114 | 96.5 | 52/52 | 100 |
|  | Group 7 | 31/36 | 85.1 | 6 | N/A | 118/118 | 100 | 28/29 | 96.5 |  |  | 41/41 | 100 |
|  | Group 8 | 32/66 | 48.5 | 20 | N/A | 191/191 | 100 | 10/27 | 37.0 | 54/60 | 90 | 32/38 | 84.2 |
|  | Group 9 | 1/1 | 100 | 6 | N/A | 6/6 | 100 | 9/9 | 100 | 5/5 | 100 | 3/3 | 100 |
|  | Group 10 | 34/122 | 27.9 | 45 | N/A | 145/269 | 54.1 | 27/124 | 21.8 | 85/173 | 49.1 | 45/127 | 35.4 |
| Presence of intrapartum protocol for healthy women | | YES | | YES | | YES | | YES | | YES | | YES | |
| Presence of protocols for healthy childbearing women | | YES | | YES | | YES | | YES | | YES | | YES | |
